# Quinine and Opium in the Cold Stage of Paroxysmal Fevers

**Published:** 1853-03

**Authors:** 


*Quinine and Opium in the Cold Stage of Paroxysmal Fevers.*—We have long thought it a desideratum in practice to find out some mode of treatment by which the practitioner may be enabled, when summoned to a case of remittent fever, *during the cold stage*, to cut short the paroxysm—to extinguish the intense febrile reaction, which usually succeeds the cold stage of our summer and autumnal diseases. When called upon to visit a patient, and we find him shivering with a chill—with blue lips and fingers—goose-flesh, shrunken surface—quick, small, feeble pulse—more or less nausea—great thirst—and in a word, laboring under the usual distressing symptoms of the cold stage of fever, we usually content ourselves with warm foot-baths, tepid drinks, etc.; but these domestic remedies serve only to hasten and augment the reaction; they do not even abridge the febrile paroxysm which must succeed the chill.

We are about to recommend measures which will meet the indications so much desired in these cases, such as we have frequently tested in hospital, and sometimes in private practice. Macintosh practiced blood-letting in the cold stage of fever; but if this treatment proved beneficial in some cases, it operated injuriously in others; and thus, by common consent, venesection in the cold stage of fever has been abandoned in the United States, as far as we know.

Full doses of quinine and opium, given in the midst of the cold or shivering stage of fever, will be found both safe and efficacious in the large majority of cases. It puts a stop to the cold or chilly stage, increases the fullness, while it diminishes the frequency of the pulse, allays, as by enchantment, the violent neuralgic pains with which the head, back, limbs, etc., of the patient are tortured, equalizes the circulation, promotes free perspiration, and rarely fails to extinguish the intense febrile reaction which, without the interposition of our art, rarely fails to succeed the cold stage of our autumnal fevers. Opium and quinine, thus administered, seem to restrain the violent action of the heart and arteries, the former of which is curbed, so to speak, by the combination, and held within its normal force and frequency. The patient passes at once from the chill or chilly, into the sweating stage—characterized by a full, soft and regular pulse, a warm, moist, and relaxed surface, absence of thirst, headache, restlessness, and the usual concomitants of intense febricity. He exchanges great suffering, pains and uneasiness, for sweet and refreshing slumbers, for the most part, from which he awakes at the end of a few hours, perfectly delighted with himself and his physician.

By this treatment, we arrest the paroxysm more certainly than if we had administered the quinine during the apyrexia; the series of morbid phenomena by which a paroxysm of intermittent fever is characterized, is broken up; and the quinine and opium appear to arrest the disease definitively, by extinguishing the germ of morbid action. The dose, in the instances recommended, should be from 20 to 25 grains of the sulphate of quinine, and from 2 to 4 grains of opium, in combination; it may be repeated in severe cases, but in diminished doses, after the first three or four hours.

Quinine and opium, given at the moment advised, are less likely to disturb the encephalon than might be suspected by those who have never ventured to try it in the cold stage of fever, to adopt a misnomer. Treated after the method above recommended, the paroxysm is broken up, and will rarely recur the second time, although we may withhold the further use of remedies. Under our plan, convalescence becomes speedily established, the patient rapidly recovering his appetite and strength. Many, and we include ourselves, venture to administer the quinine and opium during the height of the febrile excitement, with the most beneficial effects; but it must strike any reflecting mind, that if opium and quinine, given during the stage of exacerbation, exercises a sedative influence over the heart and arteries, how much more rational and easy to keep down such febrile excitement, by interposing our remedies before all the links in the chain of morbid causes which constitute a febrile paroxysm, shall become firmly united!—*N. Orleans Med. and Surg. Journal.*

